# The excretory-secretory products of *Echinococcus granulosus* protoscoleces directly regulate the differentiation of B10, B17 and Th17 cells

**DOI:** 10.1186/s13071-017-2263-9

**Published:** 2017-07-21

**Authors:** Wei Pan, Wen-Ting Hao, Yu-Juan Shen, Xiang-Yang Li, Yan-Juan Wang, Fen-Fen Sun, Jian-Hai Yin, Jing Zhang, Ren-Xian Tang, Jian-Ping Cao, Kui-Yang Zheng

**Affiliations:** 1 Jiangsu Key Laboratory of Immunity and Metabolism; Department of Pathogenic Biology and Immunology, Laboratory of Infection and Immunity, Xuzhou Medical University, Xuzhou, Jiangsu Province, China; 2 National Institute of Parasitic Diseases, Chinese Center for Disease Control and Prevention; Key Laboratory of Parasite and Vector Biology, Ministry of Health, Shanghai, China

**Keywords:** IL-10-producing B cells, IL-17A-producing B cells, *Echinococcus granulosus* protoscoleces, Excretory-secretory products, Inflammation

## Abstract

**Background:**

Excretory-secretory products (ESPs) released by helminths are well-known to regulate T cell responses in the host. However, their direct influence in the differentiation of naïve T cells, and especially B cells, remains largely unknown. This study investigated the effects of *Echinococcus granulosus* protoscoleces ESPs (EgPSC-ESPs) on the differentiation of IL-10-producing B cells (B10), IL-17A-producing B cells (B17) and Th17 cells.

**Methods:**

BALB/c mice injected with EgPSC were used to evaluate the in vivo profiles of B10, B17 and Th17 cells. In vitro purified CD19^+^ B and naïve CD4^+^ T cells were cultured in the presence of native, heat-inactivated or periodate-treated EgPSC-ESPs, and the differentiation of these cell subsets were compared.

**Results:**

In contrast to the control group, infected mice showed higher frequencies of B10, B17 and Th17 cells, and higher levels of IL-10 and IL-17A in the sera. Interestingly, B17 cells were first identified to express CD19^+^CD1d^high^. In vitro, B cells cultured with native ESPs exhibited a higher percentage of B10 cells but lower percentage of B17 and Th17 cells compared to the PBS group. Moreover, the relative expression of IL-10 and IL-17A mRNA were consistent with the altered frequencies. However, ESPs subjected to heat-inactivation or periodate treatment exhibited an inverse effect on the induction of these cell subsets.

**Conclusions:**

Our findings indicate that ESPs released by EgPSC can directly regulate the differentiation of B10, B17 and Th17 cells, which appear to be heat-labile and carbohydrate-dependent.

**Electronic supplementary material:**

The online version of this article (doi:10.1186/s13071-017-2263-9) contains supplementary material, which is available to authorized users.

## Background

Helminth parasites are highly successful pathogens, persistently infecting a quarter of the world’s population, and causing significant morbidity but rarely death [1, 2]. This is largely because that they have evolved potent and varied immune subversion strategies that facilitate evasion of host immune responses. T cell responses such as T helper 1 (Th1), Th2, Th17 and regulatory T cells (Tregs) have been extensively studied in helminth infections [3–5]. Protective immunity against helminths is thought to be partly mediated by Th2 cells, while failure to mount Th2 responses can result in immunopathology mediated by Th1 or Th17 cells. Moreover, the induction of Treg cells and the anti-inflammatory cytokines IL-10 and TGF-β plays an essential role in immune tolerance, thus prolonging the survival of parasites in hosts.

In contrast to T cells, the role of B cell subsets in helminth infection is less well understood. However, several B cell subpopulations have been shown to play essential roles in immune regulation. There is convincing evidence that following infection with *Leishmania major* and *Schistosoma mansoni* IL-10-producing B cells (B10 cells) have strong immunosuppressive activity [6–8]. This B cell subset expresses CD1d^high^CD5^+^ and produces IL-10 exclusively to suppress Th1/Th17 responses and promote the induction of Treg cells [9, 10], which have been recognized as potent negative regulators of inflammatory responses [11]. Most recently, a novel IL-17A-producing B cell population (defined as B17 cells in this study) was identified in *Trypanosoma cruzi* infection [12], and was subsequently confirmed in rheumatoid arthritis [13]. Collectively, these studies suggest that helminth parasites regulate host immune responses not only *via* the induction of effector or regulatory subsets of T cells, but also of B cells.

Excretory-secretory products (ESPs) released by helminths function as essential immunomodulators by direct exposure to the host immune system [14, 15]. Accumulating evidence has shown that ESPs induce Th2 responses by preferentially polarizing alternatively activated dendritic cells (DC) and macrophages, and diminish the inflammatory response by inhibiting Th1/Th17 responses and inducing Tregs and B10 cells [5, 16]. However, at present, it is unclear whether ESPs regulate these immune responses by directly interacting with naïve T or B cells.

The cestode *Echinococcus granulosus* is a representative helminth of medical and veterinary importance as the causative agent of cystic echinococcosis (CE). The larval stages of *E. granulosus* develop hydatid cysts in the internal organs of intermediate hosts over many years, often resulting in chronic infection. The cyst consists of two layers (germinal and laminar layers) containing the hydatid cyst fluid and protoscoleces (PSC) [17]. In this study, we focused on the response to *E. granulosus* PSC (EgPSC) infection, given that it can infect both definitive and intermediate hosts [18], and is considered to be an excellent model system for investigation of host-parasite interactions. Our previous study showed that myeloid-derived suppressor cells (MDSC) and Tregs can be induced to establish infection in mice [19]. Also, we showed that DC exposed to adult worm ESPs induced the generation of Tregs [20]. These data suggest that the parasite can downregulate T cell immune responses by interacting with DC and MDSC. Nevertheless, whether the ESPs released by the parasite directly induce the differentiation of newly identified B cell subsets, remains to be elucidated.

This study examined the effects of EgPSC-ESPs on the induction of B10, B17 and Th17 cells from CD19^+^ B and naïve CD4^+^ T cells, respectively. Our results show that native ESPs can directly promote the differentiation of B10 cells but inhibit the differentiation of B17 and Th17 cells. However, the ESPs with heat-inactivation or carbohydrate removal had an inverse effect on the induction of these cell subsets. These observations reveal an immunoregulatory mechanism by which EgPSC downregulates the inflammatory response in a heat-labile and carbohydrate-dependent manner.

## Methods

### Parasites, mice and infection

Female BALB/c mice (aged 6–8 weeks) were purchased from the SLAC Laboratory (Shanghai, China) and bred in the University facilities. The PSC were obtained by puncturing the fertile sheep hydatid cysts under aseptic conditions according to the protocols detailed in Macintyre et al. [21]. Briefly, the parasites were washed several times using sterile phosphate buffered saline (PBS), pH 7.2 containing 100 μg/ml penicillin and 100 U/ml streptomycin (Invitrogen, Frederick, MD, USA). The parasite vitality was determined by eosin exclusion. Only parasite batches exhibiting over 90% vitality were used for infection.

Thirty BALB/c mice were inoculated intraperitoneally with 200 μl of a suspension containing 2000 live PSC in PBS; 30 BALB/c mice injected with 200 μl PBS were used as the controls. All mice were bred and housed in specific pathogen-free conditions until sacrificed under sodium pentobarbital anesthesia for experimentation after 4 months.

### EgPSC cultivation and ESPs preparation

EgPSC were cultured and ESPs were prepared as previously described, with some modifications [22]. Briefly, 10,000 PSC were cultured in 2 ml of sterile PBS supplemented with 10% glucose, 100 μg/ml penicillin and 100 U/ml streptomycin (Invitrogen, USA) in 6-well plates at 37 °C in 5% CO_2_. The supernatants containing ESPs released by the EgPSC, but without the parasites, were renewed every 8 h and concentrated using Ultrafree 15 filters with a 5 kDa pore diameter membrane (Millipore, Watford, USA). EDTA (5 mM/l) and phenyl methyl sulfonyl fluoride (PMSF, 2 mM/l) were added and collected as native EgPSC-ESPs.

The protein concentration was determined using a BCA assay (Thermo Scientific, Rockford, USA). The endotoxin in the collected proteins was carefully removed using the ToxinEraser™ Removal Kit (GenScript, Piscataway, NJ, China) and then detected by the *Limulus* Amoebocyte Lysate Assay (Cambrex, East Rutherford, NJ, USA).

Alternatively, ESPs subjected to heat-inactivation (H-ESPs) were heated to 100 °C for 30 min in an electric heater. For the preparation of PSC soluble antigens (PSA), the PSC were snap-frozen in liquid nitrogen and grounded into a paste before being resuspended in PBS.

### Periodate treatment

Sodium metaperiodate-mediated modification of carbohydrates in EgPSC-ESPs was performed using a modification of Gómez-García et al. [23]. Briefly, 2 mg/ml of EgPSC-ESPs was incubated for a few seconds (*v*/*v*) with 50 mmol/l sodium acetate pH 4.5 at room temperature. The sample was divided into two to produce periodate-treated ESPs (P-ESPs) and mock periodate-treated ESPs (M-ESPs). Twenty millimolars of sodium metaperiodate (*v*/*v*) was added to P-ESPs, whereas the M-ESPs received acetate buffer without sodium metaperiodate. Both tubes were incubated for 30 min in the dark at room temperature with gentle shaking. The reaction was completed by further incubation with 100 mmol/l of sodium borohydride in PBS for 30 min at room temperature. Excess salt was removed by using Amicon Ultra Filter Units (Millipore, Billerica, MA, USA), and the protein concentration was determined.

### B cell cultivation and ESP treatment

CD19^+^ B cells from BALB/c mice were sorted positively using a mouse CD19^+^ B cell isolation kit (Miltenyi, Bergisch Gladbach, Germany); the cell purity was routinely > 90%. Purified CD19^+^ B cells were cultured in 24-well plates (5 × 10^5^ cells/well) with or without native ESPs, H-ESPs, P-ESPs, PSA, and LPS. The concentration of all stimulants was 10 μg/ml. After 72 h of culture, the cells and supernatants were collected for further analysis.

### In vitro Th17 cell polarization and ESP treatment

Naïve CD4^+^ CD62L^+^ T cells from BALB/c mice were isolated from fresh spleens using a mouse CD4^+^ CD62L^+^ T Cell Isolation Kit II (Miltenyi) with a MiniMacs Separator in combination with LS and MS columns according to the manufacturer’s instructions. The purity of the cells after separation was routinely > 85% as determined by flow cytometric analysis. For in vitro polarization of Th17 cells, purified naïve T cells were incubated in 24-well plates precoated with anti-CD3 (5 μg/ml), anti-CD28 (1 μg/ml), IL-2 (5 ng/ml), anti-IL-4 antibody (2 μg/ml), anti-IFN-γ antibody (2 μg/ml), transforming growth factor-β (TGF-β, 5 ng/ml), interleukin-6 (IL-6, 20 ng/ml), and IL-23 (10 ng/ml) (all from R&D Systems) with or without EgPSC-ESPs (10 μg/ml) [24]. After 72 h of culture, cells and supernatants were collected for further analysis.

### Flow cytometric analysis

Single-cell suspensions were prepared and filtered with a cell strainer. Surface staining was performed using the following monoclonal antibodies: anti-CD19 [clone eBio 1D3)], anti-CD5 (clone 53–7.3), anti-CD1d (clone 17–0013-80), anti-CD4 (clone L3 T4) (all from eBioscience, San Diego, CA). For intracellular staining of IL-17A and IL-10, phorbol myristate acetate (50 ng/ml), ionomycin (500 ng/ml), LPS (10 μg/ml) (all from Sigma-Aldrich, Kansa, USA), Brefeldin A (10 μg/ml, eBioscience, San Diego, USA) and monensin (2 μM, eBioscience) were added to the culture for the last 5 h before flow cytometric analysis. Anti-IL-17A (clone eBio 17B7), anti-IL-10 (clone JES5-16E3) and their isotype controls were obtained from eBioscience. For the analysis of apoptosis, B cells were collected and incubated with Annexin V-APC and 7-AAD (eBioscience) according to the manufacturer’s instructions. All stained cells were detected by the flow cytometry (Beckman Coulter, Brea, CA, USA) and the data were analyzed using Summit 4.3 software.

### RNA isolation and quantitative real-time PCR

RNA was extracted from cultured B cells using TRIzol reagent (Life Technologies, Carlsbad, CA, USA), and cDNA was synthesized from the RNA using First Strand cDNA Synthesis kit (TIANGEN Biotech, Beijing, China). Following reverse transcription, cDNA was amplified using LightCycler® FastStart DNA Master (Roche Applied Science, Penzberg, Germany) with gene-specific primers designed to amplify a portion of the coding sequences. Quantitative PCR analyses were performed in a LightCycler® 480II detection system (Roche Applied Science, Penzberg, Germany) under the following thermal cycler conditions: 5 min denaturation at 95 °C followed by 40 cycles of 30 s at 95 °C, 30 s at 58 °C, and 30 s at 72 °C. The primers were as follows: *IL-10*: Forward 5′-GCT CCA GAG CTG CGG ACT-3′; Reverse: 5′-TGT TGT CCA GCT GGT CCT TT-3′; *IL-17A*: Forward 5′-CTC CAG AAG GCC CTC AGA CTA-3′; Reverse: 5′-GGG TCT TCA TTG CGG TGG-3′; *GAPDH*: Forward 5′-CAA CTT TGG CAT TGT GGA AGG-3′; Reverse: 5′-ACA CAT TGG GGG TAG GAA CAC-3′. All experiments were performed in triplicate and the Ct values were normalized against an endogenous reference (GAPDH). The relative expressions of IL-10 and IL-17A were indicated by comparative cycling threshold (Ct) normalized against GAPDH using the 2^-△△Ct^ method.

### Cytokine analysis

Enzyme-linked immunosorbent assays (ELISAs) for IL-6, IL-10, IL-17A, TNF-α, and IFN-γ (eBioscience, USA) in culture supernatants were performed according to the recommendations of the manufacturer. Alternatively, the serum concentrations of these cytokines were evaluated using cytometric bead arrays (CBA) according to the manufacturer’s protocols (CBA™, BD Biosciences, San Jose, USA). Data were acquired with a FACSCanto II flow cytometer and analyzed using FCAP Array software v3.0 (BD Biosciences). Cytokine concentrations were calculated using standard curves.

### Statistical analysis

Data were expressed as means ± standard deviation (SD). Differences based on the data characteristics were analyzed by Student’s *t*-test, one-way ANOVA and nonparametic tests (Kruskal-Wallis H-test and Mann-Whitney U-test) using SPSS 20.0 version. *P*-values < 0.05 were considered to indicate statistical significance.

## Results

### The proportions of B10 cells were increased post-infection

**Fig. 1:**
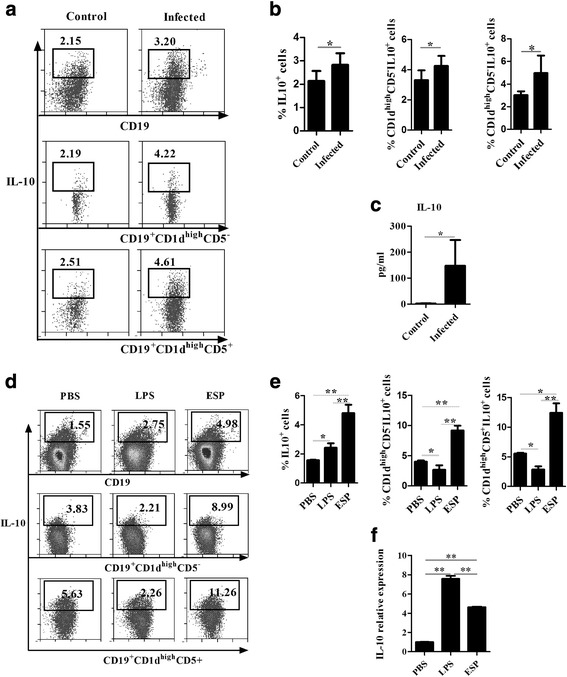
The frequencies of B10 cells post-infection and the induction of B10 cells by EgPSC-ESPs in vitro. **a** Representative frequencies of B10 cell subsets among total splenic CD19^+^ B cells in control and infected mice. Splenic cell suspensions were prepared and cultivated for 6 h. The percentages of IL-10^+^, CD1d^high^CD5^+^IL-10^+^, CD1d^high^CD5^−^IL-10^+^ among the CD19^+^ B cells were analyzed by flow cytometry. **b** Ratio of B10 cell subsets among the total splenic CD19^+^ B cells in control mice (*n* = 15) and infected mice (*n* = 15). Differences were analyzed by *t*-test. **c** Changes in serum IL-10 levels of control and infected mice, determined by CBA (BD Bioscience). Differences were analyzed by the Mann-Whitney U-test. **d** Representative flow cytometry plots of B10 cells and their subsets post-stimulation with PBS, LPS, and EgPSC-ESPs. **e** Induction of B10 cells and their subsets stimulated by PBS, LPS and EgPSC-ESPs. Differences were analyzed by one-way ANOVA. **f** The relative expression of IL-10 in cultured B cells stimulated by PBS, LPS and EgPSC-ESPs using quantitative real-time PCR. Differences were analyzed by the Kruskal-Wallis H-test. All data are expressed as means ± SD of triplicate wells in three independent experiments. Asterisks indicate statistically significant differences among the three groups. *P < 0. 05; **P < 0.01

Unlike Th1 and Th2 cells, the cytokines or subgroups of B cells associated with parasitic infection are still not well defined. To understand the role of B cells in EgPSC-infected mice, splenic cells were prepared to analyze the frequencies of B10 cells by flow cytometry, and sera were collected to detect IL-10 levels using CBA. As shown in Fig. 1a, b, the percentages of IL10^+^CD19^+^ B cells were significantly increased post-infection (*t*_(28)_ = -2.355, *P* = 0.046). Two B10 cell subsets that express CD1d^high^CD5^−^ and CD1d^high^CD5^+^ were present at increased percentages. In accordance with this, the IL-10 levels in infected sera were significantly higher than those in the control group (U_(28)_= 0.00, *Z* = -2.236, *P* = 0.036; Fig. 1c). These data suggested that B10 cells might partly contribute to the elevated IL-10 production in EgPSC-infected mice, which might be an important mechanism of immune escape implemented by the parasite.

### Direct induction of B10 cells in vitro by native EgPSC-ESPs

A previous study found that a glycoconugate-enriched fraction from EgPSC stimulated the secretion of IL-10 production by peritoneal B cells [25]. However, whether this process is directly triggered by ESPs remains to be established. To confirm the regulatory effect, CD19^+^ B cells were sorted from the splenic cells of normal BALB/c mice, and then incubated with 10 μg/ml native ESPs (the endotoxin level was lower than 0.015 EU/ml) for 72 h. As shown in Fig. 1d, e, native ESPs significantly increased the frequencies of B10 cells compared to those in the control group (*F*_(2,6)_ = 62.788, *P* < 0.0001). Accordingly, the percentages of the main two subsets that secrete IL-10 were significantly increased (*F*_(2,6)_ = 86.835, *P* < 0.0001; *χ* ^2^ = 7.261, *df* = 4, *P* = 0.007). Moreover, the relative expression of IL-10 mRNA in B cells was much higher than that in control group (*F*_(2,6)_ = 4.417, *P* = 0.022; Fig. 1f). These data suggested that native ESPs released by the parasite act directly to promote the induction of B10 cells in vitro.

### Th17 cell frequencies were elevated post-infection

**Fig. 2:**
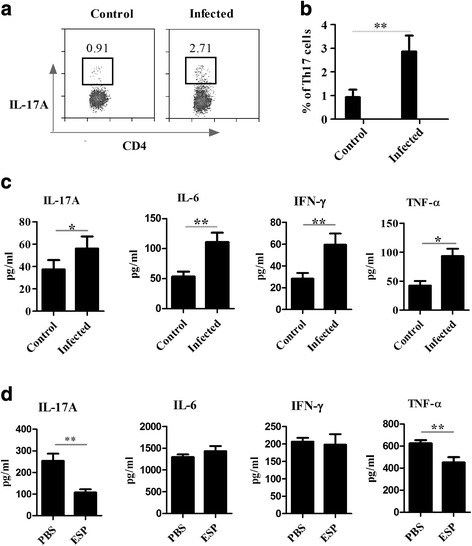
The frequencies of Th17 cells post-infection and the inhibitory effects of EgPSC-ESPs on the differentiation of Th17 cells from naïve CD4^+^ T cells under Th17-polarzing conditions. **a** Representative plots of splenic IL-17^+^CD4^+^ T cells in control and infected mice. **b** Percentages of Th17 cells among the total splenic CD4^+^ T cells in control mice (*n* = 15) and infected mice (*n* = 15). **c** Serum levels of IL-17A, IL-6, IFN-γ and TNF-α in control and infected mice determined by CBA (BD Bioscience). **d** Cytokine profiles in the culture supernatants of naïve CD4^+^ T cells after EgPSC-ESPs treatment under Th17-polarizing conditions. Differences among groups were analyzed by Student’s *t-*test. All data represent the means ± SD. Asterisks indicate statistically significant differences among the three groups. *P < 0. 05; **P < 0.01

Inflammatory responses play an important role in controlling parasite loads in hosts. To evaluate the inflammatory profiles in EgPSC-infected mice, the percentages of Th17 cells among the splenic CD4^+^ T cells and inflammatory cytokines in serum were determined. As shown in Fig. 2a, b, the proportions of Th17 cells were significantly higher post-infection than those in the control group (*t*_(28)_ = -6.919, *P* < 0.001). This is consistent with previous data obtained from the study in patients [26]. In addition, the levels of proinflammatory cytokines in serum, such as IL-17A, IL-6, IFN-γ and TNF-α, were significantly increased post-infection (*t*_(28)_ = -2.852, *P* = 0.040; *t*_(28)_ = -3.655, *P* = 0.036 and *t*_(28)_ = -9.704, *P* = 0.001 and *t*_(28)_ = -2.876, *P* = 0.039, respectively) (Fig. 2c). These data suggested an inflammatory environment in infected mice, which might, therefore, play an immunoprotective role in combating the parasitic infection.

### EgPSC-ESPs attenuated Th17 responses directly under Th17-polarizing condition in vitro

The inflammatory responses observed in mice post-infection were apparently unfavorable for parasite survival. Studies have revealed that parasite ESPs have multiple functions in subverting host immune responses [3]. The EgPSC-ESPs were, therefore, postulated to influence Th17 cell differentiation in a direct manner.

To test this hypothesis, naïve CD4^+^ T cells expressing CD62L were sorted from the spleens of normal BALB/c mice, and primed using anti-CD3 plus anti-CD28 antibodies in the presence of IL-2, IL-6, TGF-β and IL-23, as well as anti-IL-4 and anti-IFN-γ antibodies, to induce in vitro differentiation of Th17 cells. When co-incubated with EgPSC-ESPs, the frequencies of Th17 cells decreased significantly (data not shown) compared to those in the control group. The IL-17A levels in culture supernatants were reduced accordingly (*t*_(4)_ = 7.049, *P* = 0.002; Fig. 2d). These observations revealed that the EgPSC-ESPs acted directly to inhibit Th17 cell differentiation in vitro.

Other cytokines in the co-cultured supernatants were also determined. As shown in Fig. 2d, TNF-ɑ levels were also decreased after EgPSC-ESPs treatment (*t*_(4)_ = 7.785, *P* = 0.001), while the levels of the other two inflammatory cytokines (IL-6 and IFN-γ) were slightly elevated (*P* > 0.05). In addition, the levels of the anti-inflammatory cytokine, IL-10, were significantly reduced (data not shown). Collectively, these results revealed that the ESPs of EgPSC selectively regulated the expression of inflammation-associated cytokines by naïve CD4^+^ T even under Th17-polarizing conditions, reflecting a vigorous capability to regulate host inflammatory immune responses.

### The percentages of B17 cells were increased post-infection

**Fig. 3:**
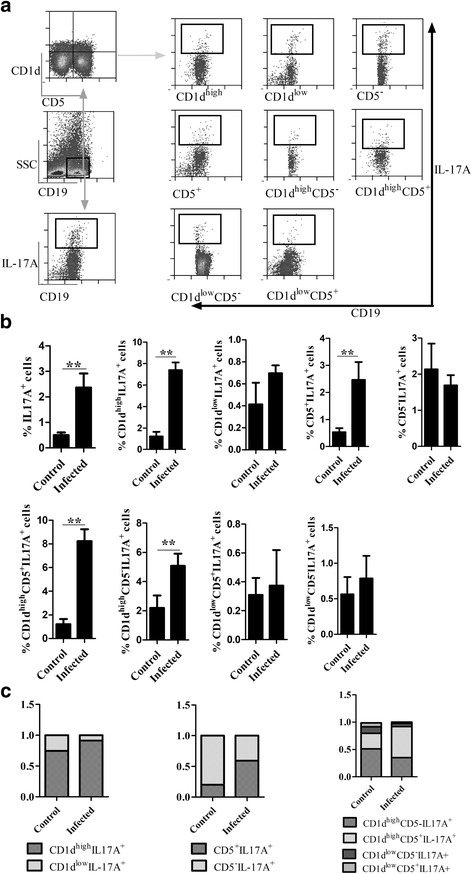
The frequencies of B17 cells post-infection and the immune-phenotype of B17 cells. Single-cell suspensions from the spleens of EgPSCs-infected and control mice were stimulated to analyze the percentages of IL-17A^+^ cells among the splenic CD19^+^ B cells by flow cytometry. The CD5 and CD1d markers that define B10 cells were used to determine the phenotype of B17 cells. **a** Example of the gating strategy shown on representative dot-plots of stimulated splenic IL-17A^+^ cells and their subsets. CD19^+^ B lymphocytes were gated, followed by the sub-gating of CD1d^+^ and CD5^+^ cells to determine the subpopulations that secrete most of IL-17A in B cells. Alternatively, IL17^+^CD19^+^ B cells were gated directly to compare the dynamic changes pre- and post-infection. **b** Frequencies of IL17A^+^, CD1d^high^IL-17A^+^, CD1d^low^IL17A^+^, CD5^+^IL17A^+^, CD5^−^IL-17A^+^, CD1d^high^CD5^+^IL-17A^+^, CD1d^high^CD5^−^IL-17A^+^, CD1d^low^CD5^+^IL-17A^+^ and CD1d^low^CD5^−^IL-17A^+^ cells among splenic CD19^+^ B cells in control and infected mice. **c** The average percentages of IL-17A^+^ producing B cell subsets among the total splenic CD19^+^ B cells in control and infected mice. Data are expressed as means ± SD (*n* = 15 mice). Differences among groups were analyzed by Student’s *t-*test. Asterisks indicate statistically significant differences between the control and infected group. *P < 0. 05; **P < 0.01

Besides Th17 cells, B cells have been recently recognized as a cellular source of IL-17A [12, 13], although the phenotype of these cells remains to be elucidated. This study was conducted to investigate the changes and the phenotypes of IL-17A-secreting B cells (defined as B17 cells) in EgPSC-infected mice. As shown in Fig. 3a, b, the proportions of B17 cells were very low in normal mice, accounting for approximately 0.5% of the splenic CD19^+^ B cells. However, the proportion significantly increased to 3% post-infection, suggesting that B cells also contributed to the higher levels of IL-17A in infected mice (Fig. 2c).

### CD19^+^CD1d^high^ was the dominant immune-phenotype of B17 cells

To characterize the immune-phenotypes of B17 cells, the markers associated with B10 cells were analyzed by flow cytometry. As shown in Fig. 3a, b, not only the percentage of IL-17A^+^ in CD19^+^ B cells, but also of its subgroups, including CD1d^high^IL-17A^+^, CD1d^low^IL-17A^+^, CD5^+^IL-17A^+^, CD5^−^IL-17A^+^, CD1d^high^CD5^+^IL-17A^+^, CD1d^high^CD5^−^IL-17A^+^, CD1d^low^CD5^+^IL-17A^+^, and CD1d^low^CD5^−^IL-17A^+^, were elevated post-infection.

CD1d^high^CD19^+^ B cells were identified as the dominant IL-17A secreting subset, accounting for approximately 80% of the population, while CD1d^low^CD19^+^ B cells were responsible for the production of only 20% (Fig. 3c), regardless of the presence or absence of infection. Moreover, CD5^+^ and CD5^−^ B cells, defined as B1 and B2 cells, respectively, both contributed to the secretion of IL-17A. To further analyze the phenotype of B17 cells, the combined expression of CD1d^high^ and CD5 was analyzed. Interestingly, B cells expressing the CD1d^high^CD5^+^ phenotype previously defined as B10 cells, were found to secrete the highest level of IL-17A, while CD1d^high^CD5^−^ also secreted considerable levels of IL-17A. The results showed that B17 cells shared a similar phenotype with B10 cells, and indicated mutual transformation of the two cell subpopulations several conditions.

### EgPSC-ESPs directly inhibited the differentiation of B17 cells in vitro

**Fig. 4:**
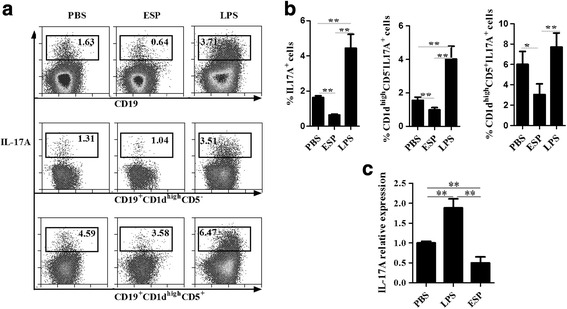
The inhibitory effects of EgPSC-ESPs on the induction of B17 cells in vitro. Splenic CD19^+^ B cells were sorted from normal BALB/c mice using a mouse CD19^+^ B cell isolation kit (Miltenyi, Germany) and cultured for 72 h in the presence of PBS, 10 μg/ml LPS, or 10 μg/ml EgPSC-ESPs. The single-cells suspensions were then collected to analyze the percentages of IL-17A^+^ and CD1d^high^CD5^−^IL-17A^+^, CD1d^high^CD5^+^IL-17A^+^ cells in CD19^+^B cells by flow cytometry. **a** Representative plots of B17 cells and their subsets after stimulation of PBS, LPS and EgPSC-ESPs. **b** Statistical analysis results. **c** Relative expression of IL-17A in cultured B cells stimulated by PBS, LPS and EgPSC-ESPs using quantitative real-time PCR. Differences were analyzed by one-way ANOVA. All data are expressed as means ± SD of triplicate wells in three independent experiments. Asterisks indicate statistically significant differences among the three groups. *P < 0. 05; **P < 0.01

Given the finding that EgPSC-ESPs inhibit the differentiation of Th17 cells, we hypothesized that ESPs influence B17 cell differentiation in a similar way. To test this hypothesis, CD19^+^ B cells were sorted and co-cultured with EgPSC-ESPs (10 μg/ml), LPS (10 μg/ml) or PBS for 72 h. The subsets of IL-17A-secreting B cells were then analyzed by flow cytometry. As shown in Fig. 4a, b, the frequencies of IL-17A secreting cells among the total CD19^+^ B cells and the two main subsets (CD1d^high^CD5^+^ and CD1d^high^CD5^−^) after exposure with EgPSC-ESPs were significantly lower than those among the corresponding populations in the control group (*F*_(2,6)_ = 54.725, *P* = 0.038; *F*_(2,6)_ = 10.722, *P* = 0.017; and *χ* ^2^ = 7.200, *df* = 2, *P* = 0.035, respectively) and LPS-stimulated group (*F*_(2,6)_ = 54.725, *P* = 0.001; *F*_(2,6)_ = 10.722, *P* = 0.003; and *χ* ^2^ = 7.200, *df* = 4, *P* = 0.010, respectively). Moreover, the relative expression of IL-17A mRNA in B cells was significantly downregulated, which was obviously lower than the levels in the control and LPS groups (Fig. 4c). Thirdly, LPS, which has been used previously to promote the generation of B10 cells, was found to significantly amplify the ratio of B17 cells and their subgroups, and might represent a positive control for B17 cell induction. Overall, EgPSC-ESPs were found to inhibit B17 differentiation in vitro, which indicated that EgPSC release products to downregulate host inflammatory responses through interacting with B cells.

### The induction of B10, B17, and Th17 cells mediated by EgPSC-ESPs was heat-labile and carbohydrate-dependent

To explore the characterization of potential molecular entities that regulate B10, B17, and Th17 induction, ESPs exposed to different treatments (H-ESPs, M-ESPs, and P-ESPs) were added into the cell culture systems, and their effects on the cell subsets were compared with native ESPs.

**Fig. 5:**
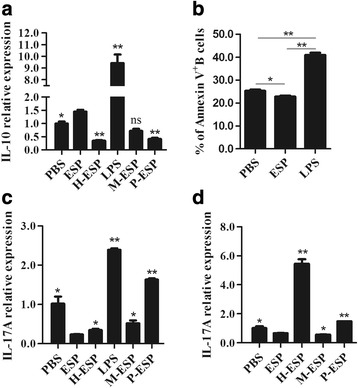
Comparison of ESPs exposed to different treatments on the expression of IL-10 and IL-17A in B and CD4^+^ T cells. **a** The relative expression of IL-10 in cultured B cells stimulated with PBS, LPS, heat-inactivated ESPs (H-ESPs), periodate treated ESPs (P-ESPs), and native ESPs using quantitative real-time PCR. M-ESPs represented a negative control to periodate treatment. **b** The percentages of Annexin V^+^ B cells following exposure to ESPs. **c** The relative expression of IL-17A in cultured B cells. **d** The relative expression of IL-17A in anti-CD3 and anti-CD28 activated CD4^+^ T cells. The differences were analyzed using one-way ANOVA. Asterisks indicate statistically significant differences between groups. *P < 0.05; **P < 0.01

As shown in Fig. 5a, the expression levels of IL-10 mRNA in B cells from the H-ESPs and P-ESPs groups were both significantly lower than those in the native ESP group (*F*_(5,12)_ = 139.710, *P* = 0.006 and *F*_(5,12)_ = 139.710, *P* = 0.004, respectively). As the negative control for P-ESPs, M-ESPs did not influence IL-10 expression in B cells (*P* = 0.180).

Given the known ability of apoptotic cells to drive IL-10 production in B cells [27, 29], ESPs were speculated to stimulate B10 *via* inducing apoptosis. However, the percentage of Annexin V^+^ B cells in the native ESP group was significantly lower than those in the PBS and LPS groups (*F*_(2,6)_ = 215.912, *P* = 0.037 and *F*_(2,6)_ = 215.912, *P* < 0.001, respectively Fig. 5b), suggesting another mechanism was associated with this process.

Figure 5c and d presented the IL-17A expression profiles in B and CD4^+^T cells. H-ESPs and P-ESPs stimulated B cells to produce higher levels of IL-17A compared to the native ESPs (*F*_(5,12)_ = 179.517, both *P* < 0.001). Moreover, P-ESPs and H-ESPs stimulated CD4^+^T cells to express higher levels of IL-17A (*F*_(4,10)_ = 183.002, *P* < 0.0001; *F*_(4,10)_ = 183.002, *P* = 0.005).

Overall, the ESPs treated with heat-inactivation and periodate inhibited B10 but promoted B17 and Th17 induction, which was inverse to those of native ESPs. These results imply that the immunoregulatory function mediated by the ESPs appeared to be heat-labile and dependent on carbohydrates.

## Discussion

Hydatid cysts, the larval stages of *E. granulosus*, are able to survive in human hosts for long periods of time, often causing chronic infection. Previous studies have revealed that the survival is prolonged by complex immunoregulatory mechanisms, such as modulated antigen-presentation by DCs and macrophages, MDSC recruitment, and Treg induction [5]. This study supports that B10, B17, and Th17 cells are also involved in the infection, which can be mediated by the ESPs released by the parasite.

B10 cells serve as a negative regulator of immune responses by the production of IL-10 and TGF-β, and also express inhibitory molecules that suppress pathogenic T cells and autoreactive B cells in a cell contact-dependent manner [28]. This study showed increased percentages of CD1d^high^CD5^+^IL-10^+^ B cells post-infection, which is consistent with the elevated IL-10 levels in infected sera. B10 cells are also activated by infection with other parasites [6–8, 11]. Notably, the frequencies of B10 cells were directly expanded after exposure to the native ESPs, suggesting that the ESPs promoted the induction of B10 cells. Furthermore, we performed additional experiments to characterize the underlying molecules in the ESPs.

Given the known ability of apoptotic cells to drive IL-10 production in B cells [27, 29], the ESPs were speculated to induce B10 cells by promoting B cell death. However, ESPs were found to slightly inhibit B cell apoptosis. Moreover, previous studies on B, T, and DC cells exposed to the live PSC also found an absence of apoptosis [22, 30]. These data suggest that the ESPs do not promote apoptosis to induce B10 cells.

Unexpectedly, both heat-inactivated and periodate-treated ESPs lost the capacity to induce B10 cells. It has been previously shown that mild periodate treatment can be used to alter the three-dimensional structure of glycans on parasite molecules [23]. Through the cleavage of bonds between adjacent carbons that bear hydroxyl groups, periodate oxidation opens sugar rings and thus destroys the integrity of carbohydrate structures. Our results revealed that B10 induction mediated by ESPs appears to be heat-unstable and carbohydrate-dependent.

As a positive stimulant, LPS was found to stimulate B10 cells *via* TLR signaling (TLR-2, TLR-4, and MyD88) [29, 31]. To exclude the disturbance of LPS, the endotoxin in the ESPs was removed. However, the ESPs still promoted the induction of B10 cells, indicating that B10 induction was not dependent on the LPS present in the ESPs.

Th17 cells play an important role in inflammatory pathology and anti-parasitic immunity [32]. In accordance with the increased levels of IL-17A in the sera of CE patients [26], this study found significantly increased Th17 cell frequencies in infected mice. This effect may beneficial to the anti-parasite immune response. Unexpectedly, the ESPs were shown to mediate direct inhibition of the differentiation of Th17 cells from naïve CD4^+^ T cells. Previous studies have revealed that ES-62 derived from *Acanthocheilonema viteae* causes MyD88 degradation to inhibit Th17 polarization [33]. Thus, it is highly likely that the Ag5 in ESPs [34], which contains the same phosphatidylcholine as ES-62, also contributes to the inhibitory effect.

In addition to Th17 cells, the percentages of B17 cells were also observed to increase significantly post-infection, and the induction of the cell population was inhibited by ESPs. To the best of our knowledge, this is the first report of B17 cells in an *E. granulosus* infection. Interestingly, similar to B10 cells, CD1d^high^CD19^+^B cells were found to predominantly express IL-17A, suggesting that a balance similar to that between Treg and Th17 cells may also exist between B10 and B17 cells, with an imbalance participating in the pathogenesis of the parasite.

Unlike Th17 cells, B17 cells are defined as a novel subset of IL-17-secreting cells, because they are stimulated *via* a mechanism that is independent of RORγt, RORα or AhR [35]. However, the induction of both Th17 and B17 cells was inhibited by ESPs, suggesting a common mechanism used to regulate IL-17A production. Notably, the expression of IL-17A was significantly upregulated following treatment with H-ESPs or P-ESPs, indicating that the regulation of IL-17 responses was heat-erratic and carbohydrate-dependent.

It is interesting that the infected mice still presented with higher levels of IL-17A compared to those of the control subjects, although the ESPs significantly inhibited Th17 and B17 differentiation. PSC somatic antigens (PSA) were speculated to promote IL-17A production in CD4^+^T and B cells; however, as shown in Additional file 1: Figure S1, similar to the effects of ESPs, PSA significantly inhibited the expression of IL-17A in these cells compared to those in PBS group. This finding suggests that PSA did not contribute to the elevated level of IL-17A production post-infection. Indeed, the contradictory observation was found to have resulted from the accumulation of monotypic myeloid-derived suppressor cells (M-MDSC) in infected mice, which were found to significantly promote Th17 and B17 induction (our unpublished data). The regulatory role of M-MDSC has also been demonstrated in mouse models of experimental autoimmune encephalomyelitis [36].

Overall, these data suggest that the IL-17-mediated inflammation response is regulated by a complex interaction between the host and the parasite. Further identification of the immunomodulatory molecules in the ESPs of EgPSCs will promote the development of novel preventive strategies against CE. However, as shown in our previous study, ESPs are a complex mixture of multiple components that are involved in a variety of biological processes [37]. Furthermore, the entities that act directly to regulate these inflammatory responses remain largely unknown.

## Conclusions

This study provides evidence that, in addition to Th17 cells, B cells also participate in dysregulated IL-17A production and enhance chronic inflammatory responses in parasite infection. Moreover, ESPs released by EgPSC significantly inhibit the proinflammatory response by direct induction of B10 cells and inhibition of B17 and Th17 cells, thereby downregulating anti-parasitic immunity. Thirdly, the effects of ESPs on the induction of these cell subsets are heat-labile and carbohydrate-dependent.

## Additional file

Additional file 1: Figure S1. The effects of PSC somatic antigens on the induction of IL-10 and IL-17A expression in B and CD4^+^ T cells. **a** and **b** present the relative expression of IL-10 and IL-17A in cultured B cells, respectively; **c** shows the relative expression of IL-17A in CD4^+^ T cells. The differences were analyzed using one-way ANOVA. Asterisks indicate statistically significant differences between groups. **P* < 0.05; ***P* < 0.01. (TIFF 1204 kb)

## Abbreviations

B10: IL-10-producing B cells
B17: IL-17-producing B cells
EgPSC: *Echinococcus granulosus* protoscoleces
ESP: Excretory-secretory product
H-ESP: Heat-inactivated ESP
M-ESP: Mock periodate treated ESP
M-MDSC: Monotypic myeloid-derived suppressor cells
P-ESP: Periodate treated ESP
SD: Standard deviation
TLR: Toll-like receptor
Tregs: Regulatory T cells
