# Supplementary figures and images for: The excretory-secretory products of Echinococcus granulosus protoscoleces directly regulate the differentiation of B10, B17 and Th17 cells
Source: Parasit Vectors. 2017 Jul 21;10:348. doi: 10.1186/s13071-017-2263-9 (PMC5520350; doi:10.1186/s13071-017-2263-9)

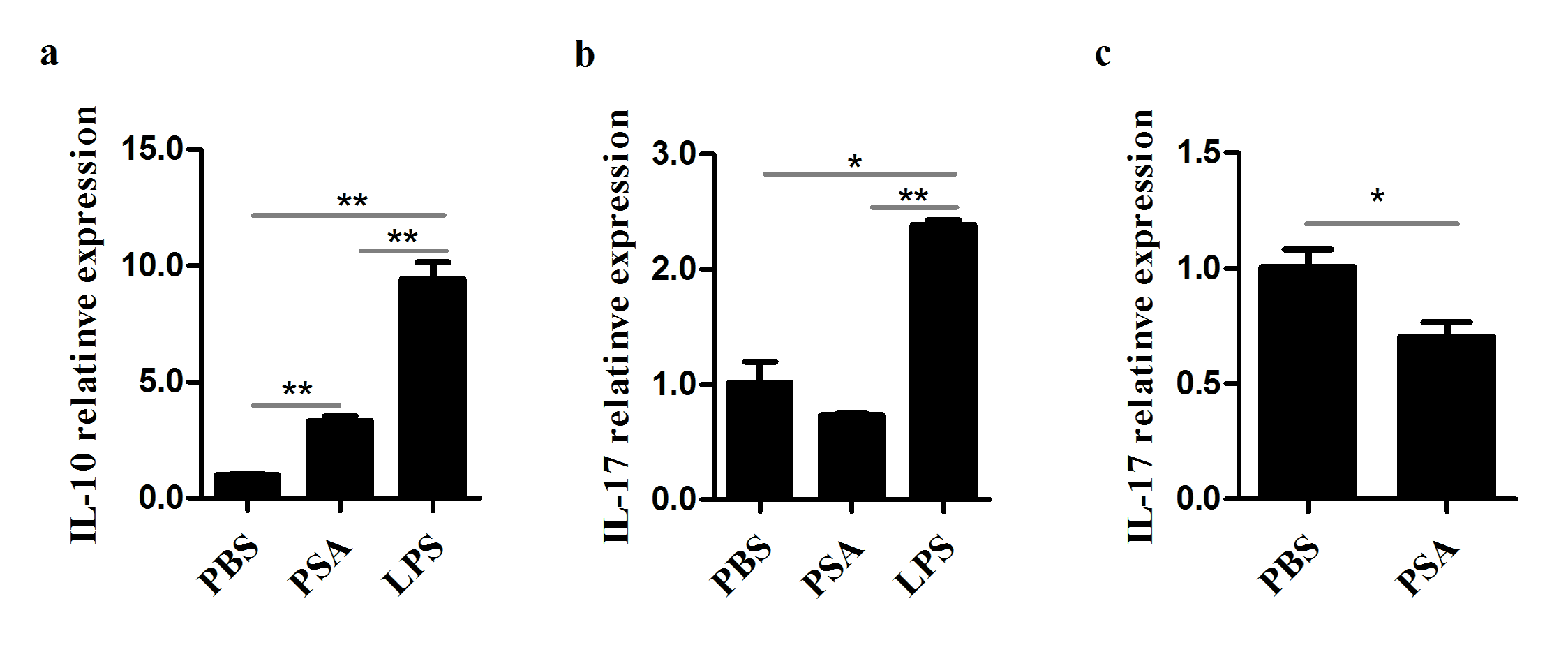

Supplement: Additional file 1: Figure S1. — The effects of PSC somatic antigens on the induction of IL-10 and IL-17A expression in B and CD4+ T cells. a and b present the relative expression of IL-10 and IL-17A in cultured B cells, respectively; c shows the relative expression of IL-17A in CD4+ T cells. The differences were analyzed using one-way ANOVA. Asterisks indicate statistically significant differences between groups. *P < 0.05; **P < 0.01. (TIFF 1204 kb) [file 13071_2017_2263_MOESM1_ESM.tif]
